# Study on spatiotemporal distribution characteristics and driving factors of carbon emission in Anhui Province

**DOI:** 10.1038/s41598-023-41507-5

**Published:** 2023-09-01

**Authors:** Jing Xu

**Affiliations:** School of Architecture and Engineering, Huangshan University, Huangshan, China

**Keywords:** Environmental sciences, Environmental social sciences

## Abstract

Carbon emission is related to global ecological security, and economic development inevitably leads to an increase in carbon emission. In recent years, as a rapidly developing province in China's economy, Anhui Province has experienced significant differences in the spatiotemporal distribution of carbon emission in different regions due to differences in development foundation, urbanization level, population size, industrial structure, etc., providing representative empirical cases for research. Based on the carbon emission data of Anhui Province before the COVID-19, this study used exploratory spatial data analysis method and Geodetector to analyze the spatial and temporal distribution characteristics and drivers of carbon emission in Anhui Province. The study found that (1) the spatial differentiation and spatial correlation of carbon emission in Anhui Province are significant, At the beginning, it shows the characteristics of "high north and low south" and "high west and low east", and then the "core–edge" structure of carbon emission becomes obvious. Carbon emission hotspot areas increase and then decrease, mainly in Hefei, Fuyang and Chuzhou City, etc. The coldspot areas are mainly located in the southern and western mountainous areas, and the degree of aggregation is decreasing year by year. (3) The level of urbanization, economic development and population size are the main driving factors of the spatial variation of carbon emissions, while the industrial structure has the least influence. And most factors produce nonlinear enhancement when spatially superimposed with other factors. (4) The high value areas of economic development, population density, secondary industry structure, and energy intensity are all at high levels of carbon emission, and a combination of factors leads to the creation of high risk areas for carbon emission. The study provides a basis for reducing carbon emission in the next stage of Anhui Province, focusing on key carbon emission areas, and sustainable development.

## Introduction

Greenhouse gas emissions have put tremendous pressure on the global environment. Environmental problems caused by global warming around the world and the increasingly prominent extreme weather are seriously threatening the survival and development of human beings^1,2^. Countries around the world are paying more attention to energy saving and emission reduction measures^3,4^. China's economy has grown rapidly and is now the second largest economy in the world after the United States. Economic growth has been accompanied by a dramatic increase in CO_2_ emissions. The special report "Global Warming 1.5 °C" released by the UN Intergovernmental Panel on Climate Change (IPCC)^5^ highlighted that China has become the world's largest emitter of greenhouse gases. In September 2020, at the 75th session of the United Nations General Assembly, China proposed the goal of carbon peaking and carbon neutrality (the "double carbon" goal), committing to peak CO_2_ emissions by 2030 and achieve carbon neutrality by 2060^6,7^. In order to achieve the peak as early as possible, in 2021, China had issued policies such as "Opinions on the Complete and Accurate Implementation of the New Development Concept for Carbon Neutrality" and "Action Plan for Carbon Neutrality by 2030" to promote the implementation of the emission reduction plan. However, due to the vast area of China, the heterogeneity of regional economic and social development leads to significant spatial heterogeneity of carbon emission^8^. Therefore, scientific understanding of the spatial and temporal distribution pattern of carbon emission and its driving factors, and analysis of regional carbon emission differences are of great significance for the formulation of emission reduction policies based on local conditions^9^.

Most of the current carbon emission studies are focused on carbon emission forecasting based on series data and panel data^10,11^, carbon emission evaluation based on regression analysis and indicator system^12,13^, carbon emission policy scenario evolution^14^, etc. The analysis of the spatial effects of carbon emission is weak, while the analysis of geospatial differences in carbon emission is an important basis for carbon emission reduction path analysis and carbon policy optimization. With the proposal of collaborative regional governance to reduce emissions, the study of carbon emission from the perspective of spatial differentiation has been increasing. Wang used a spatial analysis to analyze the spatial spillover effects of carbon emission intensity in 283 cities in China from 1992 to 2013. The overall average carbon intensity of Chinese cities is found to be decreasing and the differences are gradually decreasing, but the significant spatial clustering of carbon emission intensity is gradually increasing^15^. Azam examined the relative performance of the industry, services, and agriculture sectors in energy conservation and reduction in CO_2_ emissions in Pakistan using the “spatial–temporal decomposition” method by taken data from 2006 to 2016^16^. Numerous scholars have also conducted numerous studies on regional carbon emission and their influencing factors. Econometric and decomposition analysis models are widely used to identify the drivers of CO_2_ emissions for the study. Yoichi Kaya (1997) developed a method to analyze and predict CO_2_ emission scenarios. The IPAT identity, in its particular form was redefined as an equation relating to driving forces that determine the impact of human on the environment in the form of greenhouse gas carbon dioxide emission. Major energy and emission forecasts including IPCC special report on emissions take advantage of this equation^17^. Balsalobre-Lorente used the environmental Kuznets curve (EKC) to explore the relationship between economic growth and CO_2_ emissions in five EU countries (Germany, France, Italy, Spain, and the UK) over the period 1985–2016. The empirical results confirmed the existence of an N-shaped relationship between economic growth and CO_2_ emissions in the EU-5 countries^18^. Decomposition analysis generally decomposes carbon emission into scale effect, structural effect and technology effect^19^. Scholars have used a variety of decomposition methods for their research. For example, Xie et al. proposed a modified PDA model under the assumption of semi-disposability and decomposed the carbon emission changes in China's thermal power generation industry accordingly. They found that growth in installed capacity was seen as the largest contributor to the increase in carbon emission nationally, while a decline in capacity utilization was clearly conducive to a reduction in carbon emission^20^.

In summary, although the research on the spatial heterogeneity of carbon emission has yielded certain results, in terms of research methods, the traditional spatial methods are prone to ignore the heterogeneity of objects when analyzing objects with heterogeneity and produce mixed effects interference thus leading to errors^21^. This study proposed the use of a method for detecting spatial heterogeneity: a geographic detector for the analysis of heterogeneous objects. As an exploratory analysis tool for spatial data, the GeoDetector can not only deal with the dependent and independent variable independently, but also determine whether there is an interaction between the two influencing factors, as well as the nature and strength of the interaction, which can be applied in many fields^22,23^.

Anhui Province, located in East China, as an important part of the Yangtze River Delta economic belt, is an important energy exporting province in China and a typical area for carbon emission research. After integrating into the Yangtze River Delta economic belt, Anhui Province has experienced rapid industrialization and urbanization, but this has been accompanied by severe carbon emission and environmental problems. So "the 14th Five-Year Energy Conservation and Emission Reduction Implementation Plan of Anhui Province" clearly proposed to further improve energy conservation and emission reduction policies and mechanisms, while organizing the implementation of energy conservation and emission reduction key projects to promote a significant increase in energy use efficiency. Therefore, exploring the intrinsic drivers of carbon emission and grasping the key points of carbon emission has become an urgent issue. In this paper, we analyzed the spatial and temporal distribution and variation characteristics of carbon emission in Anhui Province before the COVID-19, and used the geographic detector method to study the intrinsic driving factors of spatial variation of carbon emission. By analyzing the correct understanding of carbon dioxide emissions in Anhui Province, we can provide reference for the government to formulate energy-saving and emission reduction policies.

## Research methods and data source

### Research methods

#### Spatial autocorrelation

According to the first law of geography, the closer things are to each other, the stronger the correlation^24^. Spatial autocorrelation analysis studies the degree of similarity between a spatial entity and its neighboring spatial entities^25^, and is divided into two types: positive and negative correlation. Positive correlation indicates that the change in the attribute value of a cell has the same trend as its neighboring spatial cells, while negative correlation indicates the opposite^26^. The global spatial autocorrelation index, which measures the spatial distribution characteristics of the whole region, and the local spatial autocorrelation index, which measures the local spatial distribution characteristics, are generally used for characterization^27^. The global spatial autocorrelation is a description of the spatial attributes in the spatial characteristics of the whole region. Determining whether there is spatial dependence can generally be tested by measuring the Moran's I index. In this paper, the global Moran's I index^28^ is used to measure the spatial correlation between the spatial unit of each city (state) in Anhui Province and the carbon emission of its neighbors, and its expression is

$$I = \frac{{n\sum\nolimits_{i = 1}^{n} {\sum\nolimits_{j = 1}^{n} {W_{ij} \left( {x_{i} - \overline{x} } \right)\left( {x_{j} - \overline{x} } \right)} } }}{{\sum\nolimits_{i = 1}^{n} {\sum\nolimits_{j = 1}^{n} {W_{ij} \left( {x_{i} - \overline{x} } \right)^{2} } } }},\quad i \ne j$$

*n* is the total number of municipalities; *x*_*i*_ and *x*_*j*_ are the CO_2_ emissions of *i* and *j* provinces, respectively. *W*_*ij*_ is the spatial weight of the region, which is used to measure the proximity relationship between the study regions. The range of *I* is [− 1, 1], greater than 0 means that the observations are positively correlated, equal to 0 means that the observations are independently randomly distributed, and less than 0 means that the observations are negatively correlated.

However, the global spatial autocorrelation can only determine the spatial correlation of carbon emission between Anhui municipalities and neighboring municipalities, and cannot identify the local spatial agglomeration characteristics. Therefore, this paper investigated the local spatial correlation patterns, i.e. the hot and cold regions of carbon emission, have the aid of the Getis-Ord Gi* index^29,30^, whose expression is

$$G_{i}^{*} (d) = \frac{{\sum\nolimits_{j = 1}^{n} {W_{ij} (d)X_{j} } }}{{\sum\nolimits_{j = 1}^{n} {X_{j} } }}$$

Standardize $G_{i}^{*} (d)$ to obtain:

$$Z(G_{i}^{*} ) = \frac{{\left[ {G_{i}^{*} - E(G_{i}^{*} )} \right]}}{{\sqrt {Var(G_{i}^{*} )} }}$$

Among them, *x*_*j*_ is the attribute value of the *j*th grid, *n* represents the total number, and the spatial weight *W*_*ij*_ is determined by comparing the distance between *i* and *j* grids with the known critical distance d. $E(G_{i}^{*} )$ is the mathematical expectation of $G_{i}^{*} (d)$, $Var(G_{i}^{*} )$ is the coefficient of variation, and a significant positive $Z(G_{i}^{*} )$ means the statistical values of the grid around the grid are higher than the mean, and the space is a high value clustering area of carbon emission, i.e. the hot spot distribution area; a significant negative $Z(G_{i}^{*} )$ means that there is low value clustering around the grid *j*, indicating that the space is a low value clustering area for carbon emissions, i.e. a cold spot distribution area, i.e. a cold spot distribution area.

#### Geodetector

Geodetector is a new statistical tool for analyzing the influencing factors behind spatial data based on spatial heterogeneity. If an independent variable has a significant effect on the dependent variable, then the spatial distribution of these two is somewhat similar^31^. Therefore, by detecting the consistency of the spatial distribution of the dependent and independent variables, the degree of influence of the independent variable on the distribution of the dependent variable can be explored. The geographic detector contains four modules: factor detection, interaction detection, risk area detection, and ecological detection^32^.

### Data source

The data on CO_2_ emissions in Anhui province are from Shan's survey^33^, which is closer to the actual situation in China than other data. The global datasets such as Carbon Dioxide Information Analysis Center (CDIAC), missions Database for Global Atmospheric Research (EDGAR) and Carbon Dioxide Information Analysis Center (CDIAC) only provide estimates for China’s overall emission or at most for a few sectors and fuels. The carbon emission inventories of 30 provinces in China were compiled using the same accounting scope, data sources, and format, ensuring internal consistency and comparability of the data. All relevant activity data, emission coefficients, and calculation codes are available for free from the China Emissions Account and Dataset http://www.ceads.net. The data of population, economy and industrial structure among the selected factors influencing the spatial differentiation of carbon emission are obtained from the Statistical Yearbook of Anhui Province. It should be noted that since the geodetector can only handle type variables, the independent variables need to be discretized based on the natural interruption point hierarchy prior to the analysis.

The spatial differentiation of carbon emission is influenced by a combination of social factors. With reference to the existing studies and the obtained data, this paper selected six factors: population density (person/km^2^), urbanization level, secondary industry structure, tertiary industry structure, economic development, and energy intensity to analyze the influence of factors on carbon emission in Anhui Province (Table 1). Among them, the population factor is one of the main drivers of carbon emission^34^, and the impact of population change on carbon emission levels under urbanization is better reflected by population density than population structure and size^35,36^. The level of economic development leads to changes in production and consumption patterns, which in turn affect the level of carbon emission^37^, so GDP per capita was chosen to characterize the level of economic development in this paper. Industrialization plays an important role in the process of carbon emission^38^, and since the economic structure of the secondary industry is a key factor in carbon emission, the ratio of the secondary industry to GDP was selected to measure the structure of the secondary industry. Considering the special industrial structure of Anhui Province, the ratio of tertiary industry to GDP was also selected to measure the structure of tertiary industry. Energy consumption in industry accounts for a larger proportion of the three major industries^39^, and total energy is a visual representation of the amount of energy consumed. This paper selected the energy consumption index per unit of GDP, which refers to the energy consumed per unit of GDP produced in a region in a certain period of time^40^.The indicator can well reflect the economic growth mode, reflect the industrial structure status, etc., and well indicate the degree of dependence on energy for economic development^41^.

**Table 1:** Spatially divergent impact factors of carbon emission.

Impact factor	Unit	Symbol	Indicator description
Urbanization level	%	ur	Urban resident population/total population ratio
Population density	Person/km^2^	PopDen	Total number of people/area
Secondary industry structure	%	is	Gross secondary industry/GDP
Tertiary industry structure	%	It	Gross tertiary industry/GDP
Economic development	Million yuan	Pgdp	GDP per capita
Energy intensity	Ton of standard coal/million yuan	ei	Energy consumption per unit of gross regional product

## Spatial and temporal distribution characteristics of carbon emission and hot spot analysis

### Spatial and temporal distribution characteristics of carbon emission

The global Moran's I index of carbon emission of Anhui Province over the years was calculated separately. For the model analysis tool, the p value indicates the probability that the observed spatial model is created by a Stochastic process. When the p value is very small, it means that the observed spatial model is unlikely to be generated from a Stochastic process. Z-score represents a multiple of the standard deviation. The higher (or lower) the z-score, the higher the degree of clustering. The statistical results are shown in Table 2, and it is found that the z-scores are all greater than + 2.58, and the p-values are all less than 0.01, indicating that the null hypothesis can be rejected, and the Moran's I index is all greater than zero and passes the significance test at the 99% level. The overall Moran's I index gradually and slowly decreased from 1997 to 2017, indicating a decreasing level of spatial agglomeration of carbon emission. However, there was a rise in 2009, with a value of 0.3361, and it lasted until 2012 before it started to continue slowly decreasing.

**Table 2:** Anhui Province's historical carbon emission global Moran's I index.

Year	1997	1998	1999	2000	2001	2002	2003	2004	2005	2006	2007
Moran's I	0.3361	0.3288	0.3247	0.3224	0.3145	0.3161	0.3083	0.3021	0.2989	0.3028	0.2869
Z	5.3656	5.2468	5.1818	5.1463	5.0280	5.0492	4.9265	4.8330	4.7843	4.8460	4.5990
P	0.0000	0.0000	0.0000	0.0000	0.0000	0.0000	0.0000	0.0000	0.0000	0.0000	0.0000

Year	2008	2009	2010	2011	2012	2013	2014	2015	2016	2017	
Moran's I	0.2778	0.3361	0.3288	0.3247	0.3224	0.2294	0.2236	0.2164	0.2013	0.1921	
Z	4.4574	5.3656	5.2468	5.1818	5.1463	3.7243	3.6359	3.5292	3.2963	3.1614	
p	0.0000	0.0000	0.0000	0.0000	0.0000	0.0002	0.0003	0.0004	0.0010	0.0016	

**Figure 1:**
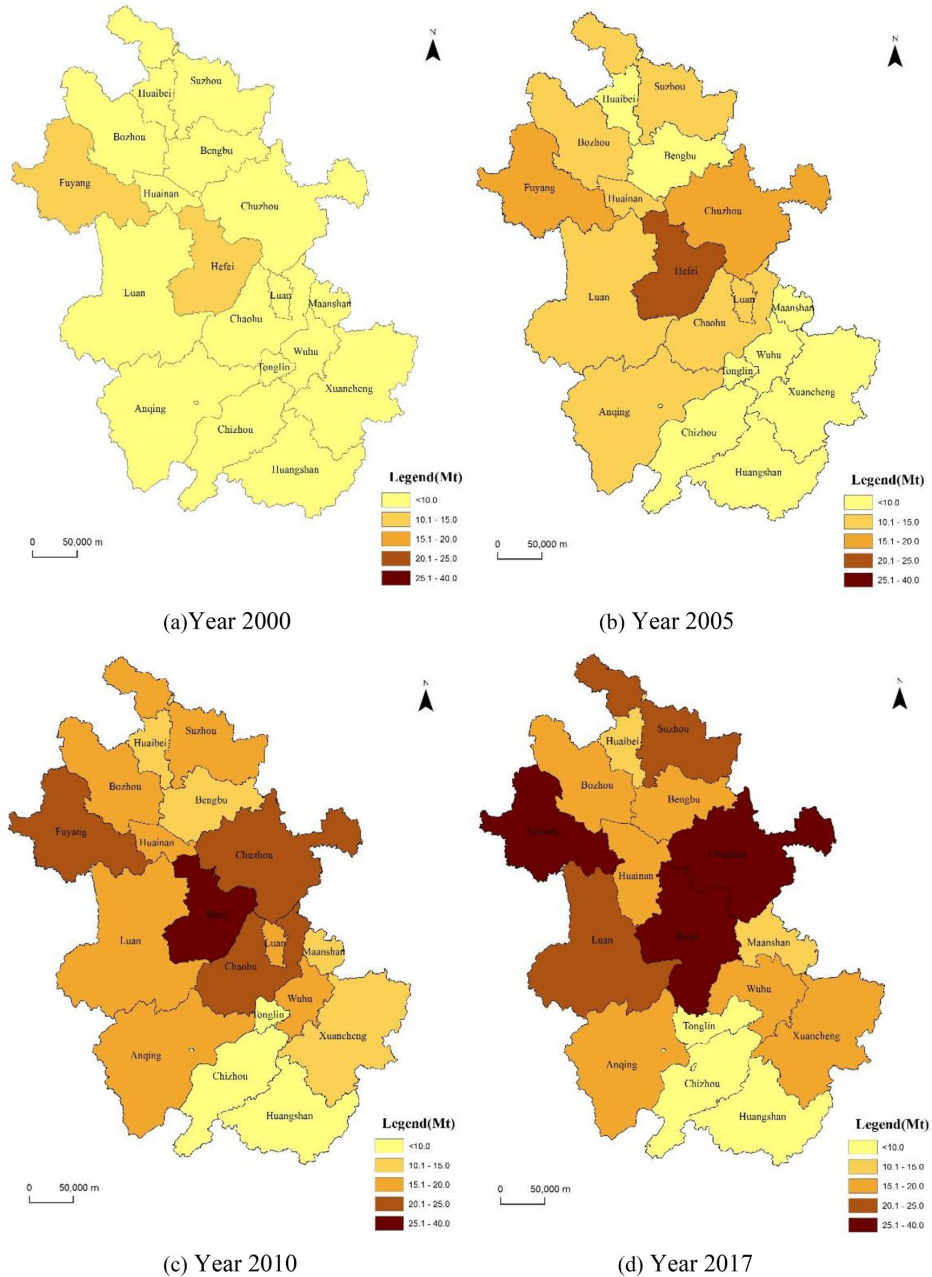
Changes in the spatial pattern of total carbon emission (Mt) in Anhui Province over the years. Software version: ArcMap 10.6, URL: https://www.esri.com/en-us/arcgis/products/indext.

In this paper, we selected four periods of sample emission data in 2000, 2005, 2010 and 2017 to plot the spatial distribution of different years, and summarized the spatial and temporal evolution patterns of carbon emission in Anhui Province. Anhui Province showed a steady and sustained growth in carbon emission from 2000 to 2017 (Fig 1). In 2000, most municipalities in the province emitted less than 10 Mt of carbon, while in 2017, most municipalities emitted more than 20 Mt of carbon. Hefei, Fuyang and Chuzhou city all had carbon emission of more than 25 Mt, and such cities are increasing day by day.

From the spatial pattern, high carbon emission cities were mainly concentrated in Hefei and Fuyang City in 2000, and Chuzhou City was added as a high carbon emitting city in 2005. From 2000 to 2005, carbon emission showed the spatial variation characteristics of "high in the north and low in the south" and "high in the west and low in the east" in the city. In 2010, Chaohu City was added as a high carbon emitting city (Chaohu was incorporated into Hefei in 2011). The carbon emission of Chuzhou and Fuyang city increased rapidly during 2010–2017, and Suzhou and Liuan f changed from low emission areas to higher emission areas, the "core-fringe" structure of carbon emission is becoming more pronounced.

It can be concluded that Hefei and Fuyang City have been the areas with high carbon emission level in Anhui Province, Chuzhou City has a clear trend of carbon emission growth in recent years, and Suzhou and Liuan City have a more stable growth of carbon emission. The reasons for the above phenomena are: (1) Anhui Province itself is rich in coal resources and belongs to the province with much traditional energy mining and consumption. As the capital of Anhui Province, Hefei City has a relatively developed economy, but its energy structure is still dominated by coal energy, and the way of energy utilization is relatively crude. Although the commodity trade structure of Hefei is dominated by products exported to foreign countries, carbon emission still need to be borne by themselves, making emissions remain high. The city of Lu'an, adjacent to Hefei, is also deeply affected. (2) Both Fuyang and Suzhou City are located in the transition zone of industrial transfer from the eastern region. With the implementation of the national strategy for the rise of central China, the central region relies on the location advantage of bearing east and west, actively developing industry and promoting industrial undertaking. The development has also contributed to the growth of carbon emission. (3) Chuzhou City is strategically located in the western Yangtze River Delta. As one of the most economically active regions in China, the rapid economic and population growth of the Yangtze River Delta has led to a dramatic increase in carbon emission in the region. The Yangtze River Delta integration strategy has also promoted the gradual transfer of resource-based industries to the Chuzhou City, and energy consumption has generated a corresponding increase.

### Spatial and temporal hotspot analysis of carbon emission

**Figure 2:**
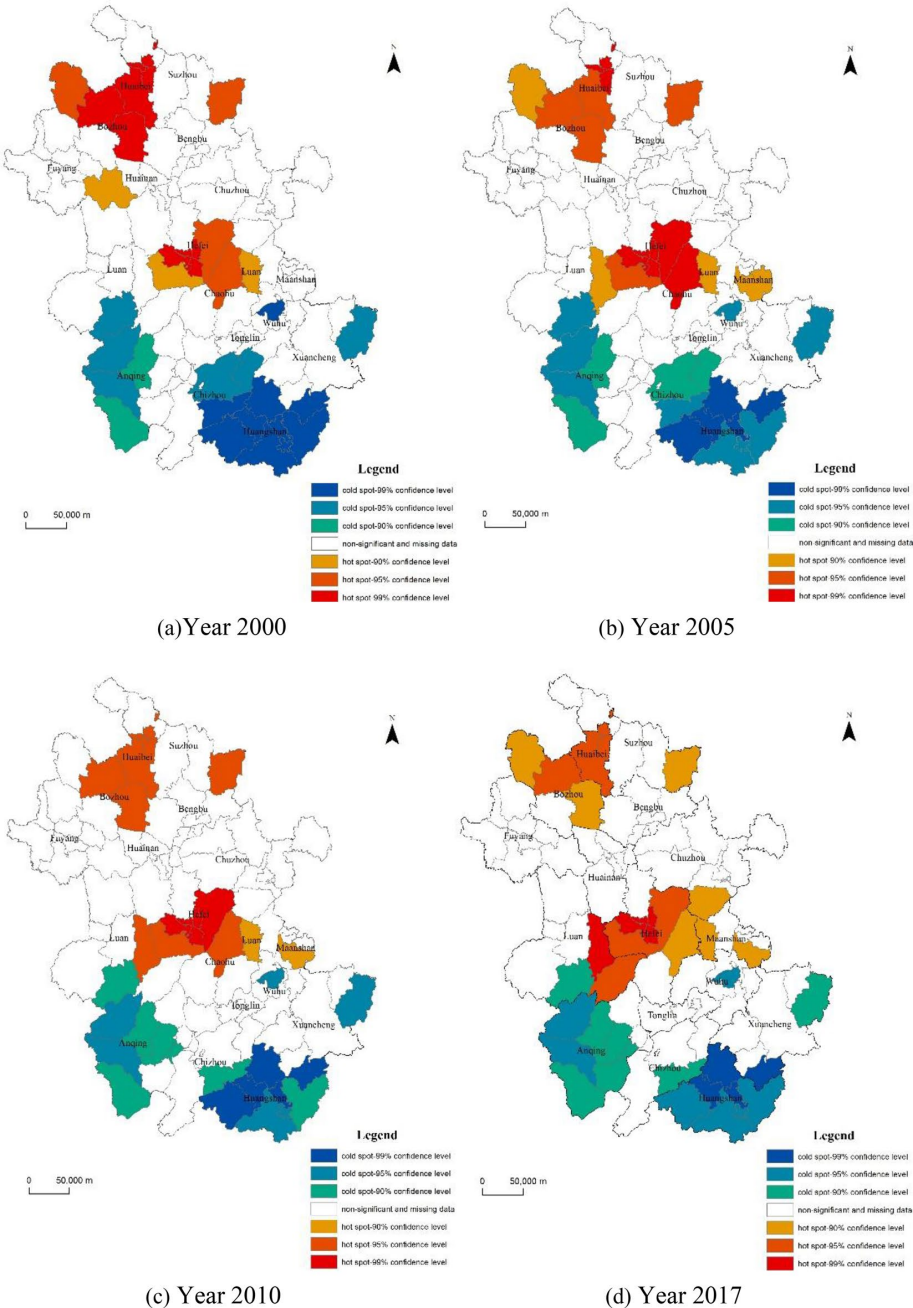
Spatial distribution of carbon emission hotspots in Anhui Province. Software version: ArcMap 10.6, URL: https://www.esri.com/en-us/arcgis/products/indext.

The spatial clustering pattern of per capita carbon emission in counties of Anhui Province is relatively fixed. High clustering (HH) and low clustering (LL) are the main types of local spatial autocorrelation. The number of districts and counties with high clustering raised and then decreased, while the number of districts and counties with low clustering gradually decreased. The carbon emission hotspots in Anhui province are mainly located in the central and northwestern parts of the province (Fig 2). The central region is dominated by the most part of Hefei, the eastern part of Lu'an City and the western part of Ma'anshan City. Since 2000, the hotspots in these areas had been expanding in size and forming continuous contiguous areas of high carbon emission. Until 2017, the area of high hotspot areas was trending downward. The northwestern part of the province, mainly the whole of Huaibei City and the northeastern part of Bozhou City, is in the hot spot area, especially before 2000, when it was a contiguous high hot spot area, and only gradually developed into a low hot spot area after 2000. The regional distribution of carbon emission cold spots in Anhui Province is roughly the same, mainly located in the southern and western mountainous areas of Anhui Province, including the whole Huangshan City, most of Anqing City, the southern part of Liuan City, some areas in the northeast of Xuancheng City and a small part in the central part of Wuhu City. However, the change in the graph also showed that the whole area of high cold spots is also slowly decreasing, indicating that the problem of carbon emission in these cities also needs to be taken seriously.

Most of the construction land and population in Anhui Province are concentrated in the central and northern regions, especially the proportion of urban land is much larger than that in the southern regions^42^. At the same time, the coal and thermal power industries associated with north-central Huainan and Huabei^43,44^ also contribute to carbon emission. With the continuous industrial restructuring and environmental pollution control in Anhui Province, the level of carbon emission is gradually being effectively controlled. The relatively clean energy consumption structure is the main reason for the low level of per capita carbon emission in cold spots, which has high forest land cover, manufacturing industries and tertiary industries that are less carbon-intensive. Since the energy consumption level in cold spots has been improved to a greater extent with economic development, but the improvement of carbon reduction technology has a lag relative to economically developed districts and counties, resulting in a decrease in the number of districts and counties that exhibit low clustering spatially.

## Analysis of the factors influencing the spatial differentiation of carbon emission

We used the carbon emission data of Anhui Province in 2017, and performed factor and interaction detection using geographic probes to reveal the role of each influencing factor and the magnitude of the interaction, and performed high-risk area detection. In geographic detectors, the spatial differentiation of the detection of Y and the extent to which the detection of a certain factor X explains the spatial differentiation of attribute Y are measured using q values. The range of q values is 0-1, The larger the q value, the more significant the spatial heterogeneity of Y. If stratification is generated by variable X, the larger the q value, the stronger the explanatory power of independent variable X on attribute Y, and vice versa.

### Factor detection and interaction detection

Have the aid of Geodetector factor detection to detect the driving force of the spatial divergence of carbon emission in Anhui Province, it can be concluded from Table 3 that carbon emissions are influenced by multiple factors, and each factor has different degrees of impact on carbon emission. The size of the influencing factors are in the following order: urbanization level > economic development > population size > energy intensity > tertiary industry structure > secondary industry structure. It can be concluded that the level of urbanization is the dominant factor in the spatial distribution pattern of carbon emission in Anhui Province, while economic development, population size and energy intensity also play an important role, and industrial structure has the least influence in the overall spatial distribution of carbon emission.

**Table 3:** Factor detection results.

Factor	PopDen	ei	Pgdp	ur	is	it
q statistic	0.1354	0.1137	0.1814	0.3040	0.0652	0.0992
p value	0.0000	0.0000	0.0000	0.0000	0.0000	0.0033

The specific meanings of the indicators in the table are shown in Table 1.

Anhui Province is still in the stage of rapid urbanization, with a population urbanization rate lower than the national average. Urbanization will continue to maintain a rapid development trend for a considerable period of time in the future. Urban infrastructure and housing construction require many high carbon emitting products, and the increase in energy consumption for residents' daily lives will also bring about new carbon emission. Anhui Province has a relatively high proportion of resource-based industries, with traditional industries dominated by energy and raw materials industries taking advantage. The energy structure is characterized by a tendency towards coal. As a developing province, the economy will continue to maintain medium to high speed development for a period of time, which means that carbon emission will continue to show an increasing trend. Urbanization has driven the agglomeration of population and other resources, leading to an increase in energy consumption through economies of scale. Moreover, the agglomeration of population has led to traffic congestion and an increase in carbon emission. The per capita energy consumption of urban residents is 3.5 to 4 times that of rural residents, and the improvement of residents' living standards will also lead to an increase in energy consumption and carbon emission. The urban and economic development characteristics of Anhui Province determine the different energy structures, industrial levels, and related impacts in different regions, and further validate the spatiotemporal distribution and hot spot distribution characteristics of carbon emission mentioned above.

### Interaction detection results

The six factors were superimposed on each other in two spaces to form 15 pairs of interactions. It can be concluded that the q-value of the interaction of each pair of influencing factors is greater than the q-value of any of the factors in the pair (Table 4). Specifically, the interaction between economic development and tertiary sector as a percentage of GDP has the largest q value (0.7836), indicating that the spatial superposition of the two plays a dominant role in the spatial distribution of carbon emission. This is followed by the ratio of tertiary industry to GDP and the level of urbanization (0.7718), and in third place by the level of urbanization and energy consumption per unit of gross regional product (0.7443). In addition, most of the other factors interacted with q values greater than 0.6, which also had a strong interaction. This also indicated that the provincial distribution of carbon emission is the result of the combined effect of various economic and social factors. The explanatory power of all interaction factors on the spatial differentiation of carbon emission in Anhui Province has increased compared to individual driving factors. The interaction between various driving factors is mainly manifested as dual factor enhancement and nonlinear enhancement.

**Table 4:** Interaction detection results.

Vriant	PopDen	ei	Pgdp	ur	is	it
PopDen	0.1354					
ei	0.4913	0.1137				
Pgdp	0.7001	0.7055	0.1814			
ur	0.6657	0.7443	0.4794	0.3040		
is	0.2966	0.7395	0.6977	0.7333	0.0652	
it	0.3716	0.6372	0.7836	0.7718	0.6481	0.09924

The specific meanings of the indicators in the table are shown in Table 1.

### Risk area detection

The six driving factors affecting carbon emission were divided into six sub-regions using the Jenks natural interruption point grading method, and the average carbon emission values of these six factors in different regions were calculated using Geodetector. The higher the regional average carbon emission value is, the region is the main impact area of this factor, i.e. high risk area.

**Figure 3:**
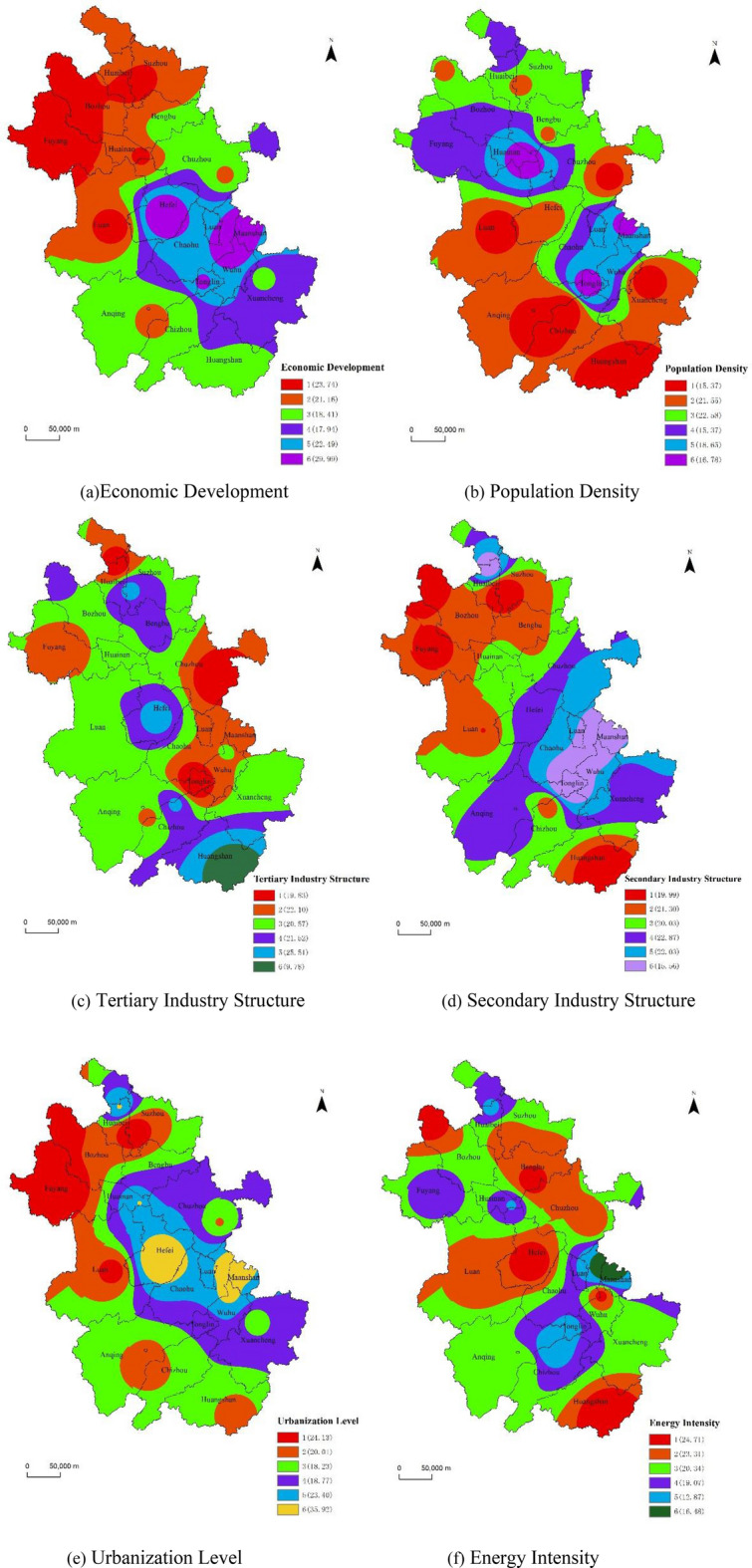
Risk factor partition and corresponding carbon emission quantity (Mt). Software version: ArcMap 10.6, URL: https://www.esri.com/en-us/arcgis/products/indext.

As shown in Fig. 3, the main influence areas of Gdp level per capita are Zone 6, including Hefei, Maanshan and Wuhu C, which are all cities with high economic development level in Anhui province. In general, the higher the level of economic development, the higher the level of carbon emission. From Fig. 2, we can see that although the economic development level in the northern and northwestern parts of Anhui Province is relatively backward, its carbon emission is not low, which indicates that the economic development mode in these regions is relatively sloppy, and the problem of high cost of resources and environment must be paid attention to in the process of economic development.

The main impact areas for population density are Zone 3 and Zone 2, which occupy most of the districts and counties in Anhui Province. In general, the higher the population density, the higher the carbon emission. Because population growth leads to an increase in domestic energy demand, human economic activity itself is one of the main triggers for the growth of carbon emission.

The main impact areas of urbanization are Zone 6, including Hefei, Maanshan and Wuhu, followed by Fuyang in Zone 1. Urbanization not only brings economic development, but also brings high energy-consuming production and living. In general, the higher the level of urbanization, the greater the carbon emission. The lower level of urbanization but higher carbon emission in Fuyang city suggests that with urbanization to a certain level, the change of living and production patterns and the accumulation of human capital can in turn suppress the growth of carbon emission.

The main impact areas of the secondary industry in the industrial structure are Zone 4 and 5, including Hefei, Chuzhou, Anqing and Liuan. The main impact area of tertiary industry is Huangshan City, the southern part of Huangshan City is developed by tourism^45^ and has very low carbon emission level. This showed that the carbon emission intensity of the tertiary industry is significantly lower than that of the primary and secondary industries. In the future, the "low-carbon + intelligent tourism" industry should be one of the key carbon reduction efforts, so as to boost the local economy while enhancing green development.

The main influence area of energy consumption level per unit of GDP is Zone 1. In general, the higher the energy consumption level per unit of GDP, the higher the dependence of economic development on energy. Hefei, Chuzhou and Bengbu are all cities with good economic development and high carbon emission. Although Huangshan City does not have high carbon emission, its industrial structure is too homogeneous, resulting in a level of economic development at the bottom of Anhui Province, and therefore a large value of energy consumption level per unit of GDP.

## Discussion

1. The analysis revealed that Anhui Province faces major challenges in environmental management, ecological construction and sustainable development. The central and northern regions are the key areas to focus on and regulate carbon emission reduction in the future, and should increase the green area and avoid the unrestricted expansion of urban construction land.
  With regard to the spatial aggregation of carbon emission, it is imperative to strengthen regional cooperation in reducing emissions, especially in high carbon emission regions. We should promote regional cooperation in technological innovation and environmental governance, and carry out joint regional governance to achieve mutual benefit and win-win situation. Cities such as Maanshan, Tongling and Fuyang should accelerate the transformation of industrial structure to energy-saving and intensive, and actively promote low-carbon and efficient production methods. Cities such as Maanshan, Tongling and Fuyang should accelerate the transformation of industrial structure to energy-saving and intensive, and actively promote low-carbon and efficient production methods. Hefei City, for example, should optimize its own industrial structure, accelerate the transformation of economic growth, continuously expand the space for production and living services, and promote the restructuring of fixed asset investment. The low-carbon zone should adhere to the transformation to energy-saving and intensive, vigorously develop low-energy-consuming, high-value-added high-tech industries and environmental industries, and actively develop the tertiary industry with the service industry as the leader. The role of different drivers on the impact of carbon emission in different regions is different, and corresponding emission reduction measures need to be taken in accordance with local conditions.
2. There are many intrinsic drivers that influence the spatial divergence of carbon emission. In this study, the factors of demographic, economic, and industrial structure were selected for analysis based on the field situation. The variety of factors can be expanded in the future to continuously improve the exploration of their influence mechanisms.
3. This study focused on the carbon emission in Anhui Province before the new crown epidemic is the spatial and temporal distribution, change characteristics and impact mechanisms, which is the limitation of this study. In the next study, the differences in carbon emission drivers before and after the epidemic are further compared and explored in terms of regional development strategies, industrial layout, and the perceived need for ecological environment, considering the analyzed impacts.

## Conclusions

Based on the carbon emission data of Anhui Province from 1997 to 2017, this paper analyzed the spatial and temporal distribution characteristics of carbon dioxide emission in Anhui Province by using spatial geostatistics and Geodetector model, and analyzed the influence of socioeconomic factors and their interactions on carbon emission. The results showed that:

1. From the perspective of spatial and temporal distribution, carbon emission in Anhui Province as a whole have shown a steady growth trend in recent years, with significant spatial divergence characteristics. At the beginning, it shows the characteristics of "high north and low south" and "high west and low east", and then the trend of "core-edge" structure of carbon emission becomes more and more obvious. Carbon emission hotspot areas first increased and then slowly decreased, mainly gathered in the middle and north of Anhui Province, including Hefei, Fuyang, Chuzhou City and other areas. The cold spot areas are mainly located in the southern and western mountainous areas, and show a gradual decrease.
2. The results of factor detection showed that urbanization level, economic development, population size, energy intensity and industrial structure are all positively related to CO_2_ emissions, among which urbanization level, economic development and population size are the main influencing factors, and industrial structure has the least strong influence.
3. The results of interaction detection showed that the growth of carbon emission is the result of a combination of socio-economic and other factors, and the effect of the two-factor interaction on carbon emission is greater than that of the single factor. The interaction effect of the tertiary structure of economic development is the largest, followed by the level of urbanization and the tertiary structure, the level of urbanization and economic development.
4. The risk area detection results showed that the high value areas of economic development, population density, secondary industry structure, and energy intensity are all at high levels of carbon emission. Only the level of urbanization has reached a certain stage of development to play a role in curbing carbon emission, and the corresponding carbon emission level is low in areas with developed tertiary industries.

Overall, the areas of Hefei, Maanshan, Wuhu, Fuyang, Chuzhou, Anqing, and Bengbu, as high-risk areas for CO_2_ emissions, are the result of a combination of various socioeconomic factors.

## Data Availability

The datasets used and/or analyzed during the current study available from the corresponding author on reasonable request.
